# Investigating human lung resilience, its evolutionary history and its relation to COPD

**DOI:** 10.1152/ajplung.00312.2025

**Published:** 2026-04-15

**Authors:** Isabelle Dupin, Maël Lemoine

**Affiliations:** 1 University of Bordeaux, INSERM, CRCTB, U 1045, Bordeaux, France; 2 Institut Universitaire de France, Paris, France; 3 University of Bordeaux, CNRS, Immuno ConcEpT, UMR 5164, Bordeaux, France

**Keywords:** COPD, evolution, resistance, smoke, tolerance

## Abstract

A subset of long-term smokers remain free of chronic obstructive pulmonary disease or lung cancer, a phenomenon termed “lung resilience.” Understanding this resilience could reveal protective mechanisms that preserve respiratory health, yet conceptual and methodological precision is required. Importantly, resilience is not merely the inverse of vulnerability, but a process that can be investigated in its own. In this review, we clarify this concept of resilience and its distinction from tolerance and resistance, and situate human lung resilience within a cross-organ and cross-species framework. We also critically assess the hypothesis that evolutionary processes may have influenced human resilience to smoke exposure. Finally, we advocate for rigorous clinical phenotyping and mechanistic approaches to dissect the interplay between genetics, physiology, and evolution. A refined conceptual framework will establish resilience as a productive paradigm in respiratory medicine, with potential to advance our understanding of disease susceptibility and to inform novel therapeutic strategies.

## Introduction

Despite high and prolonged levels of exposure to smoke, respiratory diseases such as chronic obstructive pulmonary disease (COPD) or lung cancer typically take years to develop, and not all exposed individuals are affected. A subset of smokers, referred to as “resilient smokers,” even remains healthy according to functional, clinical, and radiographic criteria (1). This phenomenon, known as “lung resilience” (1, 2), may contribute to the maintenance of respiratory health (3). Understanding the general mechanisms that underlie the various degrees of human ability to remain free of lung disease is a promising avenue for identifying protective physiological traits. The conceptual framework of resilience offers valuable insights for understanding smoke-related lung diseases. As proposed by Soriano and Polverino (2), investigating COPD in nonsmokers, as well as the hypothesis that COPD occur in exceptionally resistant survivors to earlier smoke-related diseases, may help discriminate among core mechanisms of lung injury and defense from those that are specific to smoke exposure. However, several conceptual and methodological aspects deserve clarification and will be critically discussed later.

## Defining Lung Resilience

In a common populational sense, resilience is a comparative concept: some individuals are more resilient (=less vulnerable) than others, whatever the causes, physiological or otherwise (3). In the broad physiological sense, lung resilience involves a combination of partially overlapping mechanisms against any challenge for lung health. It theoretically includes resistance, tolerance, and resilience (in the strict sense). Resilience in the strict sense defined here is triggered by sensed specific events that caused some loss of function resolved with time through a variation in response. In resistance and tolerance (see Ref. 4), there is no loss of function, but, respectively, annihilation or neutralization of the event, either through passive properties (e.g., resistance by epithelial barrier impenetrability) or through active processes (e.g., basal mucus production). For instance, although continuous mucus production generally provides resistance, damage-induced proliferation of epithelial stem cells provides resilience in the strict sense; both overlap to some extent with the effect that more mucus production involves less need of proliferation capacity, and vice versa, as illustrated by the compared evolutionary resilience strategies of the lung which has evolved mucociliary clearance, and the gut epithelia, which has evolved rapid turnover of epithelial cells (5). The impairment of any of these components (sensor, resolution, amplitude of the variation in response, passive properties, or active processes) can account for a loss of resilience (in the broad sense).

### Placing Human Lung Resilience In A Cross-Organ And Cross-Species Perspective

The resilience of the human lung to environmental challenges has been proposed to be remarkable (2), yet robust scientific evidence supporting this claim is limited. First, it has been hypothesized that “human lungs have evolved to be the body’s most resilient organ against the harmful effects of inhaled smoke” (2), but whether the lung is truly more resilient than other barrier organs, such as the intestine or the skin, regarding specific challenges remains to be demonstrated. Indeed, even if mucus production is more intensive in airways than in gut epithelium, comparison only makes sense in the light of the challenges these different tissues face. Second, whether certain species are more lung-resilient to some specific perturbations has not been conclusively shown. Also, is the human lung uniquely adapted to particle exposure when compared with, for instance, animals exposed to high levels of dust? An evolutionary perspective may help clarify this.

### Questioning Evolutionary Perspectives On Lung Resilience

In general, evolution brings innovations that confer beneficial adaptations but often come with vulnerabilities, creating the potential for disease (6). A typical example in lung science is a variant of interleukin 13, which promotes a stronger Th2-mediated immune response to pathogens, yet predisposes individuals to a higher risk of developing asthma (7), in a classical evolutionary framework of antagonistic pleiotropy. Similarly, some immune-related gene variants linked to strong inflammatory responses may raise COPD risk (8). Once advantageous against lethal infections, they now trigger excessive inflammation after smoke exposure (9), in a classic case of environmental mismatch (when an organism has not yet evolved in response to a sudden environmental change, dysfunction ensues). Antitrypsin deficiency, a specific form of COPD, illustrates this: in the heterozygous state, the S and Z alleles of α1-antitrypsin enhance inflammation and may have conferred an advantage against respiratory infections in the past (10). However, beyond this case, large genome-wide association studies (GWAS) do not strongly support an infection-inflammation trade-off in COPD. Instead, they mainly identify variants involved in lung development, extracellular matrix pathways, histone deacetylase binding, Wnt and SMAD signalling, MAPK cascades, and transmembrane receptor serine/threonine kinases (11). Some of these regions are also linked with idiopathic pulmonary fibrosis but show opposite effects, supporting the concept of divergent remodeling trajectories arising from aberrant repair pathways (12, 13).

However, evolutionary perspectives on lung resilience must be approached with caution, in particular the claim that human resilience to smoke results from evolutionary adaptation (2) (see CHECKLIST FOR AN EVOLUTIONARY CLAIM ON RESILIENCE). This hypothesis, which presumes that some observed “good” effect necessarily results from direct selection, is based on adaptationist views that have now been abandoned (14). This evolutionary scenario overlooks alternative hypotheses, including exaptation, which is an accidentally beneficial effect of a trait selected for another effect, and genetic drift, a stochastic process of change in allele frequency (14). Such nonadaptative processes could have played a role, for example, during the intense glaciation period (~0.8–0.9 Ma), which coincides with a dramatic population bottleneck for humans (15). Indeed, genetic drift is much more likely to happen in small populations, whereas intense cold itself, rather than exposure to fire, is sufficient to create sustained evolutionary pressure on the evolution of lung resilience to cold as well as to other challenges.

In this review, we also emphasize the fact that the evolution of health must not be conflated with the evolution of diseases. Although an evolutionary approach has already introduced more or less overlapping categories of evolutionary mechanisms of pathogenesis since (16), a general evolutionary classification of the counterpart of mechanisms of health (17) has never been proposed as a systematic explanation to heterogeneous susceptibility to various diseases as far as we know. More specifically, general considerations about the evolution of resilience in relation to health and disease emphasize the multilevel and complex nature of resilience (18), but do not introduce systematic classifications of forms of resilience with reference to their roles in various diseases. However, distinctions should be made between resilience mechanisms for a given disease that have likely evolved *1*) against that specific disease [e.g., Asian and African elephants present multiple copies of TP53, which is likely to have evolved for a specific anticancer effect in a big, long-lived mammal (19)], *2*) against a generic challenge that includes that specific disease [e.g., dark skin pigmentation has likely evolved in humans against many dangers, including, but not specifically, skin cancer (20)], or *3*) have just happened to protect against a disease without having evolved against it, specifically or generally. One should also remember that late-onset diseases (defined by postreproductive peak of incidence) cannot be counterselected in most species because they have no impact on reproductive fitness (21), but that protective genes can be selected, for instance, in rare cases of social species where postreproductive individuals may significantly improve reproductive fitness as humans are according to the so-called “grandmother hypothesis” (22).

**Figure 1:**
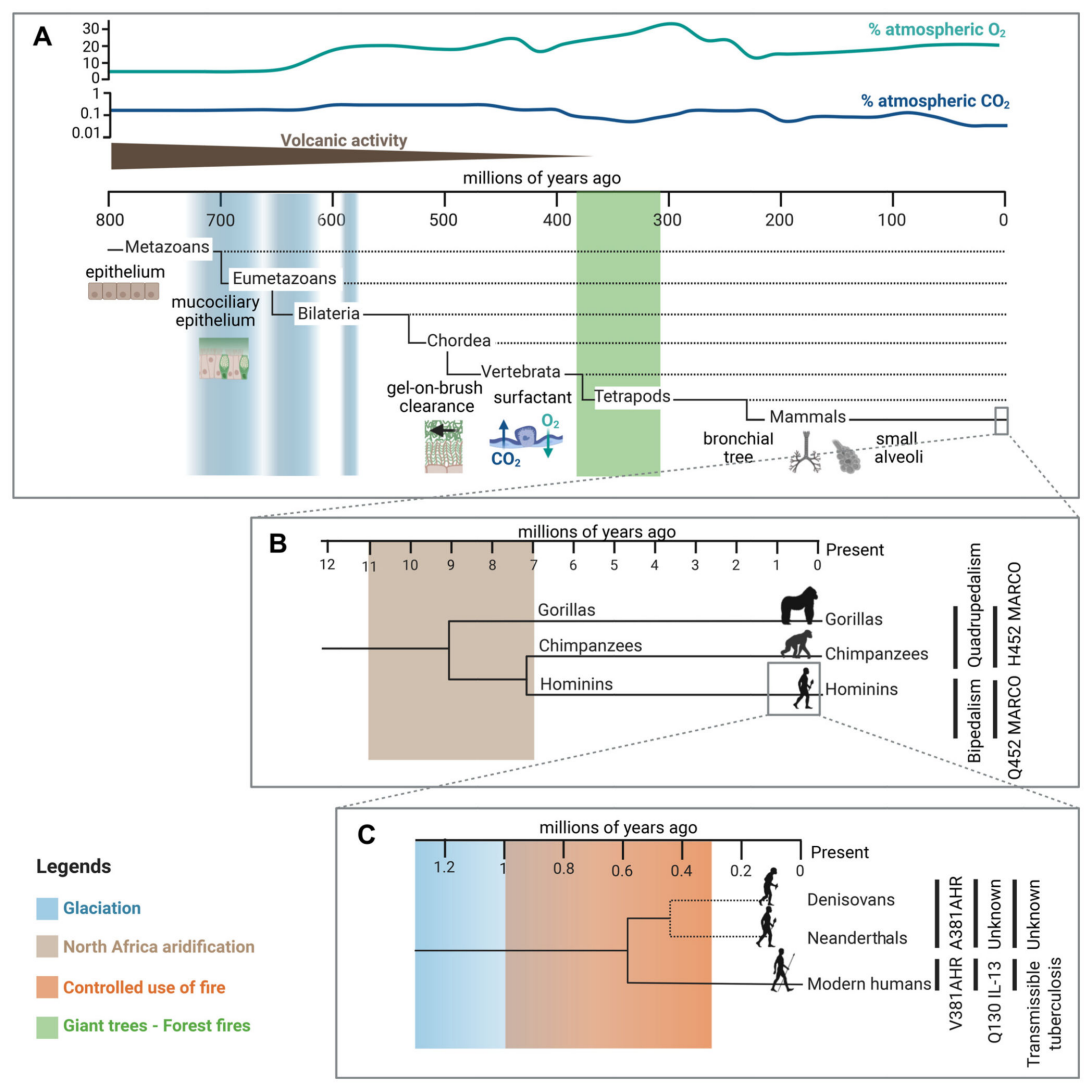
Evolutionary insights on lung resilience. Simplified phylogenetic trees of Metazoan, Hominid, and Hominin evolution, with key inventions in relationship with lung resilience/tolerance/resistance mapped either on the right or directly in the tree or within it. Time spans shown are the last 800 million years (*A*), 12 million years (*B*), and 1.3 millions of years (*C*). Major environmental changes are indicated with colored bars. Modern Human: *Homo sapiens*, Neanderthal: *Homo neanderthalensis*, Denisovan: *Homo denisova*. Figure created with a licensed version of BioRender.com.

Most mechanisms contributing to lung resilience are complex, multifactorial traits that evolved gradually over extended evolutionary timescales. Mucociliary clearance provides an example of such an evolutionary trait. It likely evolves from mucociliary epithelia, common in Eumetazoans (23) (Fig. 1A). The “gel-on-brush” clearance system (24) consists of a viscoelastic mucus mesh-like gel layer, made of water, globular proteins, and high-molecular-weight mucin polymers transported over a periciliary layer composed of membrane-bound mucins. The flow is generated by motile cilia at the apical side of epithelial cells. The biophysical properties of this polymer gel, including viscoelasticity, osmotic pressure, adhesion, cohesion, and friction, exhibit a highly nonlinear dependence on concentration, thereby critically determining its rheology and transportability (25). It emerged in aquatic vertebrates, before terrestrial life and air breathing (26; Fig. 1A). As a highly efficient system for preventing the deposition of inhaled particles on epithelial surfaces, it became prominent with the evolution of air breathing. This system is confined to the airways and provides compartmental resistance by mechanically trapping inhaled particles and ensuring their active removal through coordinated ciliary beating.

At the site of gas exchange, resistance is ensured by a fundamentally distinct system: pulmonary surfactant. Unlike mucus, surfactant does not form a gel but a thin, lipid-rich interfacial film composed of phospholipids, neutral lipids, and proteins, already present in primitive fishes and lower tetrapods (27; Fig. 1A). As lungs became increasingly subdivided in terrestrial vertebrates, evolving in mammals into a complex bronchial tree ending in alveoli (Fig. 1A), the surfactant retained its resistive role while gaining importance in reducing alveolar surface tension. In mammals, this function is enhanced by the enrichment in disaturated phospholipids (28). The surfactant sustains surface tensions well below equilibrium values, thereby preventing alveolar collapse and minimizing mechanical stress during breathing. Such efficiency relies on a dynamic equilibrium in which the alveolar film undergoes compression-induced collapse and respreading while maintaining functional stability (29).

Thus, mucus and surfactant represent two evolutionarily conserved but biophysically distinct resilience systems that dynamically protect different anatomical compartments of the respiratory tract. They primarily function as resistance mechanisms. In addition, injury or infection may enhance mucus and surfactant secretion, ciliary activity, providing features of resilience in the strict sense, by helping restore airway and alveolar integrity. Together, they protect not only against smoke but also against dehydration and a wide range of inhaled particles, suggesting that selective pressures other than smoke exposure have contributed to shaping lung resilience. Those include fluctuations of oxygen and carbon dioxide concentration in the atmosphere and the oceans, global temperature variations, geological processes, and pathogen infection, among others (30) (Fig. 1A). As some resilience mechanisms, such as pulmonary surfactant, fulfill several functions simultaneously, additional selective pressures likely contributed to their evolution, including the mechanical constraints of alveolar stability.

Nonetheless, some studies have examined possible genetic adaptations of the lung to environmental perturbations. In particular, two major events may have shaped lung resilience, not specifically in humans, but in a broader clade of hominins (Fig. 1, B and C). The first is the aridification of North Africa (7–11 Ma), which increased exposure to dust, pollen, and endotoxins, potentially selecting for Macrophage Receptor with Collagenous Structure receptor variants enhancing innate immunity (31). The second is the controlled use of fire in confined environment (1–0.3 Ma), which increased exposure to toxic particles (32, 33). However, this may have favored traits improving tolerance rather than resilience (in the strict sense): in some of the human species using fire, a variant of the aryl hydrocarbon receptor with attenuated response to chemicals present in fire smoke is observed in Sapiens but absent in Neanderthals and Denisovans (34; Fig. 1C). Whether this reflects true selection for traits conferring tolerance to smoke-filled environments remains a matter of debate. Moreover, health impacts of fire use may have broader adverse consequences, notably facilitating host susceptibility to pathogen infection and facilitating its transmissibility. By promoting social aggregation around fires, thus increasing physical proximity among individuals, and transiently altering bacterial clearance and macrophage-mediated local immunity, the use of fire has been proposed to play a role in the emergence of tuberculosis as a transmissible disease (35; Fig. 1C). These findings underscore the need to consider not only biological factors but also cultural and social behaviors as potential contributors to the development of human lung resilience, or, conversely, increased vulnerability to environmental insults.

Although most evolutionary hypotheses have focused on events that may have shaped lung traits across the hominin lineage, it is also essential to consider patterns of variation among human populations. Examples include anatomical variations in the size and shape of the thorax within the sapiens lineage, which seem to be climatedependent (36, 37) and altitude (hypoxia)-dependent (38). They are important to investigate, in an evolutionarily informed perspective, as they may influence responses to environmental exposures and susceptibility to respiratory diseases.

### Checklist For An Evolutionary Claim On Resilience:

General requirements for evolutionary hypotheses [after (39)]:

- Evidence that variations in traits are transmitted to the progeny (heritable variation, e.g., genetic),
- evidence that these variations confer a comparative advantage in survival or reproduction (differential survival or reproduction),
- identifiable, plausible, multiple, and contextualized selective pressures.

Specific requirements in the context of health and disease:

- The natural history of a disease is distinct from the natural history of mechanisms of health that are involved against that disease (e.g., specific pressure of smoke vs. generic mechanism of mucociliary clearance).
- All selective pressures on one mechanism of health should be considered and given weight according to evidence, and not according to the researcher’s focus (e.g., cancer researchers should not consider the evolution of efficient immunity variations against cancer as the cause of their emergence when these variations may as well have protected against infection).
- The presence of a health-related trait does not involve its having been selected (when beneficial) or its resulting from antagonistic pleiotropy or environmental mismatch (when deleterious): many such traits result from genetic drift or exaptation (i.e., they were selected for one advantage but later provided another one).

Specific requirements for evolutionary hypotheses on resilience mechanisms:

- Qualify the trait: tolerance, resistance, or resilience? Traits of tolerance and resistance are more likely to be simple traits with easier scenarios to imagine, but thinner evidence that they have been selected. Resilience (stricto sensu) is more likely to be a complex trait with a long evolutionary history, many forms across the Tree of Life, more evidence of selection, but a harder specific scenario to establish.
- Evidence of sufficiently long exposure to the risk, especially if recent in human history (for humans).
- Comparatively lower frequency of the genes conveying protection in closely related primates (for humans).

### Articulating The Concept Of Lung Resilience With Copd Pathogenesis

**Figure 2:**
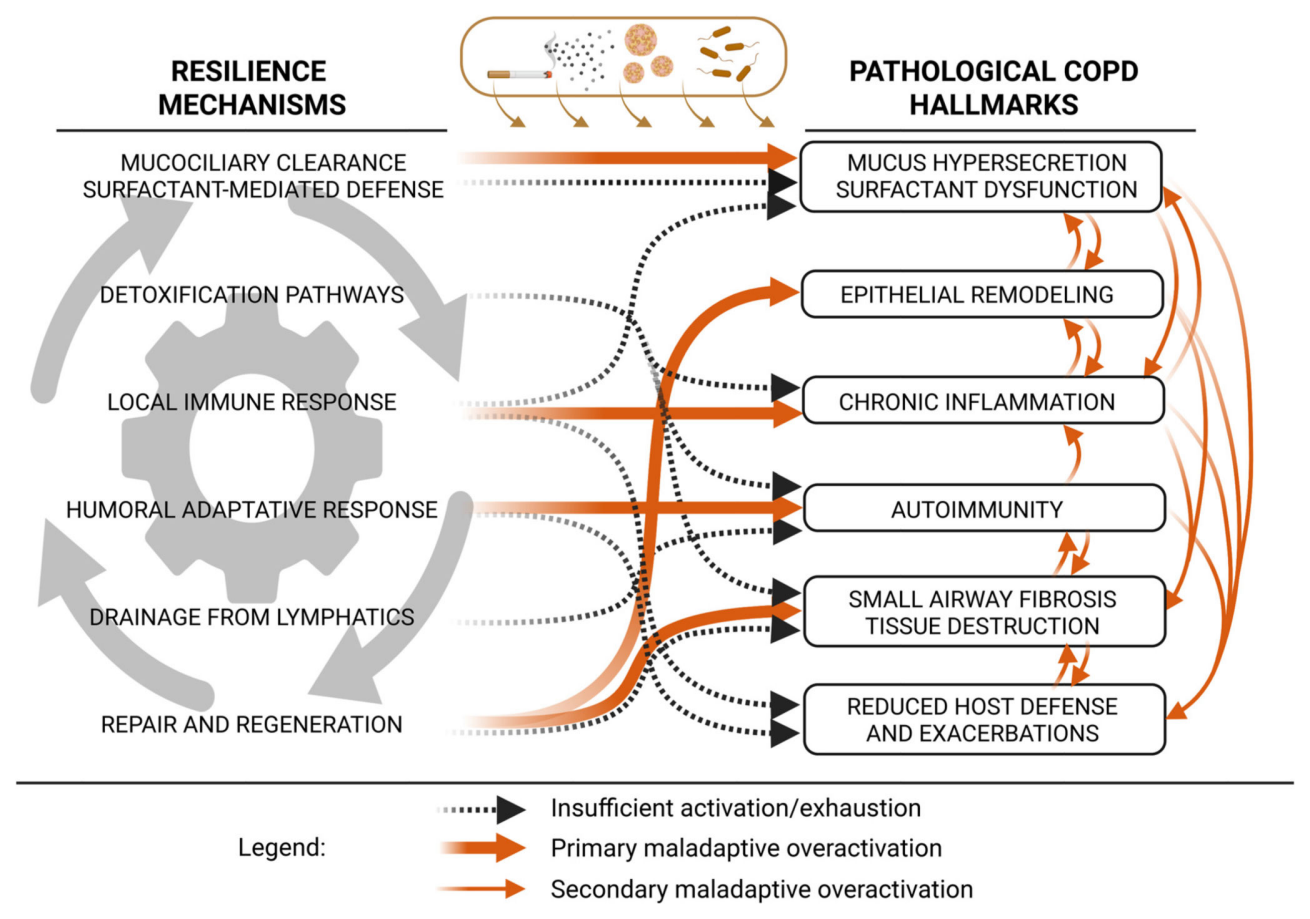
Interactions between lung resilience mechanisms and pathological hallmarks of chronic obstructive pulmonary disease (COPD). Resilience processes interact and cooperate with each other, as illustrated by the gray cogwheel and arrows. Chronic exposure to cigarette smoke and recurrent infections can lead to insufficient or exhausted resilience mechanisms (dashed lines) or to their maladaptive overactivation (solid orange arrows). Secondary maladaptive overactivation (lighter orange arrows) further contributes to the development of key COPD pathological hallmarks. Figure created with a licensed version of BioRender.com.

In the light of these general evolutionary and phylogenetic perspectives, lung resilience can thus be much more accurately and usefully conceptualized. In the lung, resilience relies on numerous processes, including mucociliary clearance, surfactant-mediated defense, detoxification pathways, local innate and adaptive immune responses, humoral immunity, lymphatic drainage, and repair and regeneration, that together maintain tissue integrity and function. A key challenge, however, is to articulate how these physiological mechanisms relate to the pathological hallmarks, such as those of COPD. We now clarify this link through three complementary scenarios (Fig. 2):

*1*) Insufficient activation of a resilience mechanism or its exhaustion leads to pathological hallmarks.

A simple example is impaired repair and regeneration of alveolar type 2 epithelial cells, which contributes to defective alveolar regeneration and ultimately alveolar destruction. In addition to these alterations, different modes of cell death, including apoptosis, necroptosis, and ferroptosis, as well as dysregulated autophagy and cell exhaustion processes such as cellular senescence, play critical roles in amplifying epithelial injury and limiting effective tissue repair (40). However, we also emphasize that isolated dysfunction of a single resilience mechanism is rarely sufficient to generate COPD pathology on its own (Fig. 2). Rather, alveolar wall destruction arises from the combination of limited alveolar epithelial regenerative capacity and the accumulation of senescent cells (which worsens with age), increased oxidative stress (reflecting impaired detoxification pathways), and chronic inflammation (e.g., CD8^+^-mediated cytotoxicity), that further enhance epithelial and endothelial cell death.

*2*) Some features interpreted as disease may represent conditionally adaptive responses.

Exposure to cigarette smoke can induce respiratory epithelial cells and submucosal glands to increase mucus production as a protective response, manifesting clinically as mucus hypersecretion. Similarly, chronic exposure to cigarette smoke can drive sustained activation of immune cells, resulting in chronic inflammation. Thus, pathological features may emerge from adaptive mechanisms that become persistent or excessive (Fig. 2).

*3*) Overactivation of a resilience mechanism can produce secondary pathological hallmarks.

Excessive mucus production, while initially protective, can overwhelm the mucociliary clearance system, leading to mucus accumulation, formation of mucus plaques and plugs, and increased susceptibility to infection and inflammation, favoring epithelial remodeling, chronic inflammation, and exacerbations (Fig. 2).

Importantly, resilience mechanisms may be simultaneously insufficient in one region of the lung and overactivated in another. For example, repair and regeneration may be impaired in alveolar regions while being hyperactivated in small airways, contributing simultaneously to emphysema and small-airway fibrosis. Resilience mechanisms also evolve over time: for instance, alveolar type 2 epithelial cells show reduced regenerative capacity with age (41).

Resilience processes do not operate independently from each other: they interact extensively and often cooperate. For example, local immune responses and mucociliary clearance work together to eliminate inhaled particles: some alveolar macrophages and their phagocytosed particles are transported to the conducting airways, where mucociliary transport ultimately enables swallowing or expectoration. More broadly, resilience mechanisms display a high degree of functional redundancy, meaning that several pathways can compensate for one another while using distinct processes. This redundancy helps explain why individuals can show different “vulnerability profiles,” why the system is generally robust, and why the system tends not to collapse abruptly but instead enters a state of escalating maladaptive overactivation across multiple levels, as each mechanism of resilience may react in turn to another’s overactivation, feeding the overactivation further. However, in particularly vulnerable individuals, deficits in specific mechanisms can reduce this redundancy, causing earlier breakdown of resilience. For instance, decreased ciliary function compromises mucus transport and predisposes to chronic infection and airway dilation characteristic of bronchiectasis. Conversely, excessive or altered mucus production, especially in combination with environmental exposures such as cigarette smoke, can contribute to airflow limitation typical of COPD. Finally, the degree of temporal, spatial, and functional overlap between the different mechanisms defines a hierarchy of functions to preserve, and may help to explain why some lung regions are more vulnerable than others.

A better understanding of this topic is essential, as several hallmarks of COPD, such as mucus overproduction, are often viewed strictly as pathological and even as causal contributors to disease progression [e.g., associations between mucus plugs and mortality (42)]. However, they may represent adaptive or compensatory responses to particulate exposure or infectious challenges. A deeper understanding of these processes is therefore essential to determine whether they should indeed be targeted directly or whether they primarily signal an upstream defect in other resilience mechanisms. In some cases, it may be more effective to target the consequences rather than the mechanism itself. For example, in the context of mucus hypersecretion, an ongoing trial aims at assessing TMEM16A modulators to improve mucus hydration and clearance (43). In other cases, however, directly reinforcing endogenous resilience mechanisms, such as repair and regenerative pathways, may represent a transformative therapeutic strategy, with the potential to shift treatment approaches from slowing down disease progression to actively restoring lung structure and function (44). In COPD, although the proliferative potential of progenitor cells may be impaired (45), some studies nevertheless support the persistence of a regenerative capacity. Normal epithelial stem cell niches remain detectable in COPD lungs (46), potentially sustaining repair processes. An expansion of epithelial cells in a regenerative state has been described in early stages of the disease, followed by a decline with disease progression (12), suggesting that endogenous repair responses are present, at least in moderate stages of COPD. Importantly, a regenerative capacity has been demonstrated in both COPD lung and COPD mouse models (47), reinforcing the concept that lung repair may be achievable through stimulation of epithelial progenitor proliferation. Such regenerative approaches could involve modulation of pathways such as Wnt/β-catenin (48, 49) or FGF10 signaling (47). Despite these advances, although individual mechanisms have been relatively well studied, the overall organization and integration of lung resilience mechanisms remain poorly understood. Bridging this gap requires appropriate experimental systems, including animal, ex vivo models, and advanced in vitro models.

### Advocating For Observational And Mechanistic Approaches On Lung Resilience

Addressing these questions will indeed require both clinical studies across diverse populations, with clearer measurable endpoints, and experimental approaches linking genetic background and gene regulation to functional outcomes. Regarding clinical studies, we think that the phenotype described by Oh et al. (1), characterized by a smoker phenotype with normal spirometry and lung function decline, no history of exacerbation during 1 year, as well as no radiographic abnormalities, provides a solid methodological framework for identifying individuals less susceptible to smoking-related damages. Building on this, observational studies should incorporate the longitudinal characterization of lung function trajectories (50) and their relationship with lung function states during childhood, including catch-up and growth failure patterns (51). Recent developments in nonirradiating lung magnetic resonance imaging also make it possible to assess structural and functional metrics over time, thereby complementing spirometry by providing spatially resolved information on tissue integrity, ventilation, and perfusion (52). In parallel, integrative multiomic analyses (e.g., genomics, epigenomics, transcriptomics, proteomics, metabolomics) could help define molecular signatures distinguishing resilient from susceptible individuals. Recent large-scale singlenucleus transcriptomic studies of COPD lung tissue, coupled with spatial transcriptomics and plasma proteomics, illustrate this potential by identifying pathologic cell states and matched circulating biomarkers, linked to clinical phenotypes (12). A key challenge will be to connect and integrate these heterogeneous datasets to identify cellular components, cross talks, and molecular pathways associated with clinical lung resilience.

Understanding lung resilience also requires experimental systems that can capture its spatial and temporal complexity, while supporting chronic exposure to environmental challenges. Because resilience mechanisms operate over diverse time scales and interact with each other, animal models remain indispensable. They provide an integrated context in which immune, epithelial, and mesenchymal populations interact over extended periods, enabling insights such as the protective role of α-Klotho in lung injury (53). Ex vivo systems, particularly precision-cut lung slices, offer preserved architecture and regional heterogeneity while allowing controlled perturbations (54). Although they have a limited lifespan in culture and do not allow to study immune cell recruitment, they are well suited for investigating localized responses and short-to mid-term adaptations. In vitro systems, including advanced cocultures, organoids, and lung-on-chip models, facilitate mechanistic studies (55). Increasing model complexity generally enhances physiological relevance and notably, robustness: for example, immune-competent lung-on-chip systems display greater resistance to viral infection than the same system lacking immune cells (56), and airway organoids show higher tolerance to pollutants compared with cell lines (57). Beyond these advantages, organoids provide powerful tools to investigate how genotypes and gene regulatory networks modulate cellular responses relevant to lung resilience (58) and to understand the evolution of the different cell types (59). They can be easily combined with an evo-devo perspective to uncover how evolutionary variations in developmental programs contribute to differences in lung response to environmental challenges. Such approaches have been recently successfully used to identify human-specific intestinal epithelial features and explore their functional relevance (59). However, most in vitro systems still struggle to reproduce the long time scale over which resilience typically operates. Combining these complementary platforms will therefore be essential to understand lung resilience and its limits.

To conclude, we strongly support the claim that resilience deserve as much attention as vulnerability does. However, a more rigorous conceptual framework that *1)* clearly distinguishes resilience (stricto sensu) from tolerance and resistance, *2*) explicitly articulates them in a broader concept of resilience (largo sensu), and *3*) considers the complexity of the evolutionary processes involved is required to ensure that resilience emerges as a productive and meaningful paradigm in respiratory research. This has medical implications, as it could help us to understand the susceptibility to lung diseases and to identify potential targets for medical intervention.
